# Standardization of Education of Patients With Cancer in a Low- and Middle-Income Country: A Quality Improvement Project Using the *Cancer and You* Booklet

**DOI:** 10.1200/JGO.19.00118

**Published:** 2019-07-08

**Authors:** Olivier Habimana, Vestine Mukeshimana, Albertine Ahishakiye, Protais Makuza, Vedaste Hategekimana, Clemence Muhayimana, Emmanuel Dushimana, Cyprien Shyirambere, Jennifer Haley, Sandra Urusaro, Stephanie Kennell-Heiling, Lori Buswell

**Affiliations:** ^1^ Butaro District Hospital, Ministry of Health, Butaro, Rwanda; ^2^ Partners in Health/Inshuti Mu Buzima, Kigali, Rwanda; ^3^ Dana-Farber Cancer Institute, Boston, MA; ^4^ University of Pennsylvania, Philadelphia, PA

## Abstract

**PURPOSE:**

The Butaro Cancer Centre of Excellence is the first comprehensive referral cancer center in Rwanda and at its inception did not have a standardized patient education program. Partners in Health/Inshuti Mu Buzima and the Rwandan Ministry of Health conducted a quality improvement project to increase patient knowledge by implementing a standardized oncology education program using picture-based and culturally appropriate materials designed for patients with cancer in low- and middle-income countries.

**METHODS:**

Four Rwandan nurses were trained to provide patient education using the *Cancer and You* education booklet created by Global Oncology. A pre- and post-test design was used to evaluate patients’ knowledge of cancer, treatment, and management of adverse effects. Nurses administered a posteducation questionnaire in Kinyarwanda to determine patients’ level of satisfaction with the education session and booklet. The four nurses were interviewed at the completion of the project for their feedback. A total of 40 oncology patients were included in the pilot project, of which 85% reported completing primary school or less.

**RESULTS:**

On average, participants improved 19% (95% CI, 13.9% to 24.1%; standard deviation, 16%) from pre- to postevaluation, demonstrating a significant increase in knowledge (*P* ≤ .001). Nearly all patients (97.5%) reported that they were either satisfied or very satisfied with the education program. Oncology nurses gave positive feedback, highlighting that it was helpful to have a standard tool for education with descriptive illustrations for those patients with low literacy.

**CONCLUSION:**

Implementation of a standardized patient education program demonstrated a statistically significant increase in patient knowledge and a high level of satisfaction among patients and nurses. The project serves as an example for other low- and middle-income countries looking to standardize oncology patient education.

## INTRODUCTION

The GLOBOCAN database 2018 report, maintained by the International Association of Cancer Registries, predicts that cancer will be the leading cause of death in the 21st century; one in eight men and one in 10 women will develop cancer in their lifetimes.^1^ It is predicted that the number of cancer cases globally will increase by 70% during the next 20 years.^2^ Whereas current data suggest that high-income countries and low- and middle-income countries (LMICs) have similar rates of cancer incidence, LMICs have the largest number of new cancer cases annually as a result of the global population distribution.^1^ Thus, the need for oncology-specific patient education materials that are specifically designed for LMICs has become paramount.

The importance of patient education in cancer care has been widely established. Chelf et al^3^ have indicated that patients reported improved outcomes in nearly all patient education intervention studies reviewed to date. A systematic review by Rutten et al^4^ highlighted specific benefits of patient education in cancer treatment: improved satisfaction in treatment, increased patient participation in decision making, decreased anxiety, and increased ability to cope with diagnosis and treatment. As a result of the overwhelming benefit, standards of safe chemotherapy administration outlined by ASCO and the Oncology Nursing Society guidelines recommend patient education before each treatment and highlight that education should be performed at a level commensurate with the patient’s learning ability.^5^

**CONTEXT**

**Key Objective**
Is a standardized oncology education program using picture-based and culturally appropriate materials designed for patients with cancer in low- and middle-income countries effective in increasing patient knowledge of cancer, treatment, and management of adverse effects?
**Knowledge Generated**
Using a standardized oncology patient education tool, nurses assessed patients’ knowledge and conducted patient education for newly diagnosed oncology patients. On average, participant pre- to post-test scores increased 19% (95% CI, 13.9% to 24.1%; standard deviation, 16%), showing a significant increase in knowledge (*P* ≤ .001). Oncology nurses felt that the standard tool for education, which included descriptive illustrations, was helpful, particularly for those with low literacy.
**Relevance**
The standardized oncology patient education program using the *Cancer and You* booklet provides an example for other low- and middle-income countries aiming to increase patient knowledge for newly diagnosed patients with cancer in low-literacy and -resource settings.

The Butaro Cancer Centre of Excellence (BCCOE) is the first comprehensive referral cancer center in Rwanda and at its inception did not have a standardized patient education program or patient education materials that discussed the basics of cancer or treatment information. Rwanda’s literacy rate for adults older than age 15 years is between 65% and 70%.^6,7^ Current patient education practices of oncology nurses include a verbal discussion between the patient and/or family, nurse, and physician regarding diagnosis, treatment course, adverse effects, and the goals of treatment.

In 2013, a nonprofit organization, Global Oncology, in collaboration with oncology experts, designed a picture-based and culturally appropriate cancer education booklet that follows guidelines and suggestions from the National Cancer Institute and low literacy education specialists.^8,9^ Illustrations designed for the booklet were piloted by Global Oncology in multiple LMIC sites to ensure that materials were culturally appropriate.^8^ The booklet, titled *Cancer and You*, was originally drafted in English and later translated into native languages. Implementation of the use of *Cancer and You* demonstrated a nearly 40% increase in knowledge about cancer and chemotherapy adverse effects in an academic teaching hospital setting in Haiti.^10^

On the basis of these results, BCCOE, in collaboration with Partners in Health/Inshuti Mu Buzima, Rwandan Ministry of Health, Dana-Farber Cancer Institute, and the University of Pennsylvania, implemented a quality improvement project to standardize cancer education for patients and their families. The aim of the project was to increase the patients’ understanding of cancer, treatment, and management of adverse effects within the context of a formal patient education program using the *Cancer and You* booklet. Secondary goals of this project included evaluating the relationship between *Cancer and You* and adult oncology patients’ knowledge, with a focus on both written and illustrated material, and determining patients’ preferences regarding the timing of receiving the education materials, teaching sessions, and overall satisfaction of this approach. Butaro Hospital administration and Partners in Health/Inshuti Mu Buzima approved the quality improvement project.

## METHODS

### Sampling and Setting

BCCOE is part of a rural district hospital, a secondary care facility in northern Rwanda.

### Nurse Training for Patient Education

Four oncology nurses were selected as patient educators in this quality improvement initiative. Nurses completed a 1-day training program on how to provide patient and family education using the *Cancer and You* education booklet, which was translated into Kinyarwanda by a professional translator. Nurses were trained to educate patients using four sections from *Cancer and You*: basic information about cancer, common adverse effects of chemotherapy, criteria for seeking medical attention, and instructions for caring for people with cancer at home. Training for these nurse educators included lecture, demonstration, and role playing. Each nurse had the opportunity to practice and receive feedback from the trainer and peers on his or her delivery of the standardized patient education. Time was also allotted for group discussions and a question-and-answer period. The section of the booklet that focuses on recording adverse effects was not used for the purposes of this project. Nurses were instructed to provide education and evaluation in a private room with the patient and his or her family member, if present.

Forty patients were selected from the adult oncology ward at BCCOE to participate. Patients were included if they were coming for the first or second cycle of chemotherapy. The first two to four patients on the daily chemotherapy list who met enrollment criteria were chosen to prevent selection bias. No more than four patients were selected per day because of nurse staffing constraints for carrying out patient interviews and conducting patient education sessions. All patients provided informed consent to be interviewed and to participate in the patient education program. Patients were enrolled from October 2016 to February 2017.

### Assessment

Nurses collected patient demographic information, including sex, age, self-reported literacy, highest level of education attained, and occupation. To assess a patient’s knowledge, a 20-question pre- and post-test design developed by the study team was used. After completing the post-test, patients were asked to complete a survey that evaluated their level of satisfaction with the booklet and the preferred timing of receiving *Cancer and You* booklet education. Nurses read evaluation and survey questions to all patients regardless of self-reported literacy and recorded patient answers verbatim. All educational sessions and evaluations were completed in Kinyarwanda. In addition, the four nurses who performed the teaching sessions were interviewed at the end of the project to provide feedback on the implementation process. They were asked to comment on successes, challenges, and future recommendations regarding the standardized patient education implementation for BCCOE.

## RESULTS

Data from the pre- and post-tests were entered into an Excel database by a BCCOE oncology nurse who was not directly involved with patient education and assessment implementation to provide consistency in data management throughout the project. Results were analyzed in Excel and STATA software (STATA, College Station, TX; Computing Resource Center, Santa Monica, CA). Descriptive and bivariable analyses were used to describe patient demographics and pre-and post-test analysis. Seven questions on the pre- and post-test were not related to material covered in the *Cancer and You* booklet and were therefore not included in the analysis of patient knowledge.

### Demographics

A total of 40 patients completed the pre- and post-test, of which 29 (72.5%) were female and 11 (27.5%) were male. Median age was 48 years with a range of 43 to 60 years. Seventy percent of patients self-reported being literate and 85% of patients reported completing primary school or less. The primary occupation of the group was self-reported as farming (79%; Table 1).

**TABLE 1:**
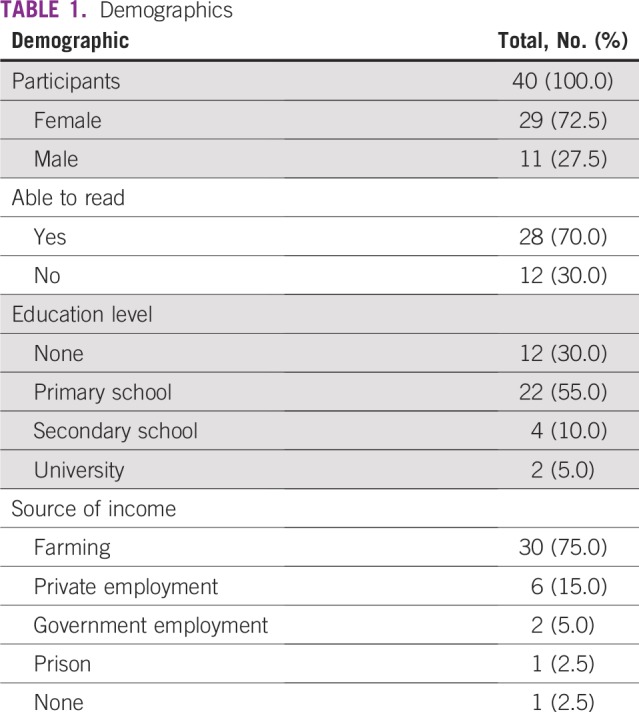
Demographics

### Pre- and Post-Test Scores

**FIG 1:**
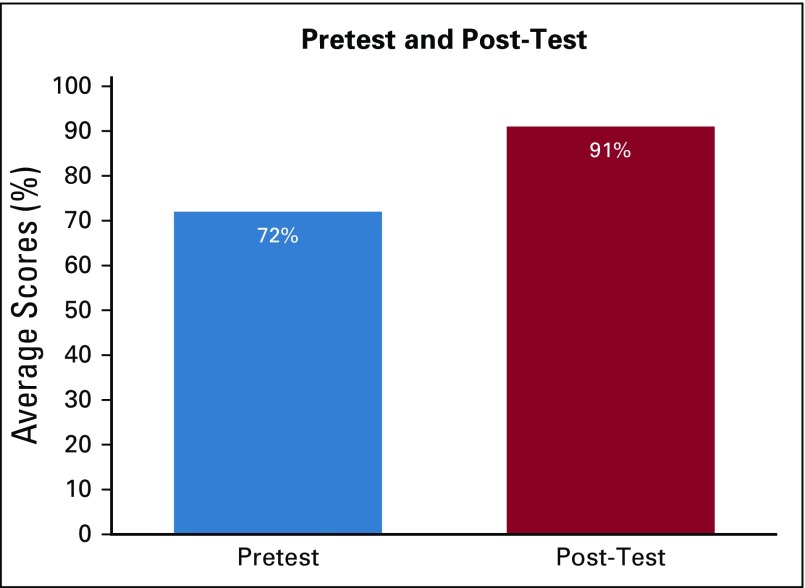
Average pretest and post-test scores.

Overall average pretest score was 72% (range, 31% to 100%; standard deviation [SD], 16.8%) and average post-test score was 91% (range, 54% to 100%; SD, 10.5%; Fig 1). On average, participants improved 19% (95% CI, 13.9% to 24.1%; SD, 16%) from pretest to post-test, which was statistically significant (*P* ≤ .001).

### Patient Evaluation of *Cancer and You*

**FIG 2:**
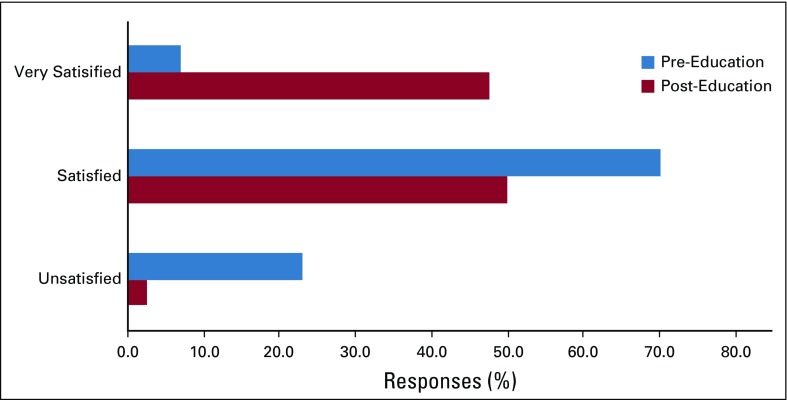
Level of patient satisfaction with the *Cancer and You* booklet.

**FIG 3:**
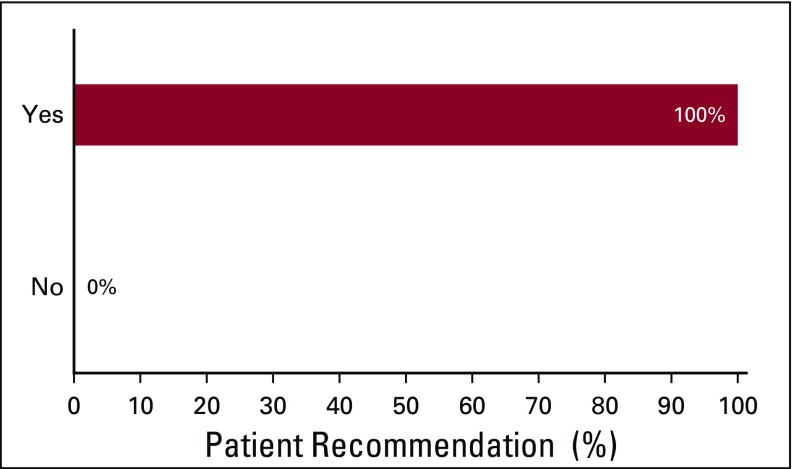
Patient recommendation for giving the *Cancer and You* booklet to future chemotherapy patients.

**FIG 4:**
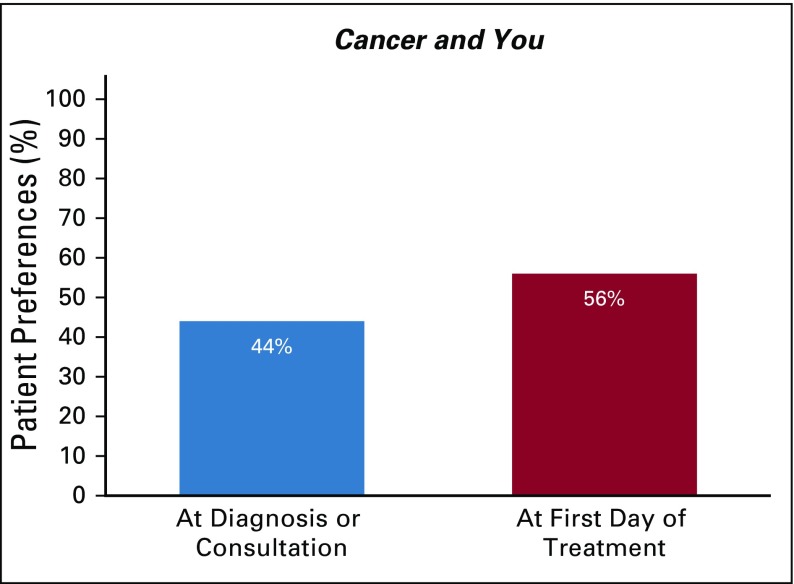
Patient preferences for time to initiate *Cancer and You* booklet education.

Before the implementation of the use of the *Cancer and You* booklet, approximately 25% of patients were unsatisfied with their education about cancer, treatment, and treatment adverse effects. After patients were educated by the nurse using the *Cancer and You* booklet, nearly all patients (97.5%) reported that they were either satisfied or very satisfied with their education (Fig 2). All patients (100%) recommended giving this booklet to other patients receiving chemotherapy (Fig 3). In addition, 100% of respondents (39 of 40; one patient did not respond) desired to take a copy of the booklet home with them to share with their families. Responses to the future timing of patient education using the booklet were divided. Forty-four percent of patients indicated that they would prefer to receive the booklet and education at the time of diagnosis, whereas 56% preferred to receive these on their first day of treatment (Fig 4).

### Nurse Facilitator Feedback

After completing all interviews, nurses were asked to provide feedback on their experiences using the *Cancer and You* booklet. Nurses reported that it was helpful to have a standard tool to use for education. Nurses stated that the illustrations in the booklet also allowed patients who could not read text to still engage with the material and ask questions about cancer treatment adverse effects.

Nurses reported that the main challenge with implementing the use of the *Cancer and You* booklet was creating time for patient education. In general, they reported that it took almost 2 hours to administer the demographic survey, pretest, deliver the education, and administer the post-test. Forty minutes of the 2 hours was spent delivering the educational material. Nurses felt that it was difficult to spend this amount of time on patient education for one individual, particularly on days when nurse staffing was low and other nursing duties, such as chemotherapy mixing, chemotherapy and medication administration, wound dressing changes, and ward rounding, all needed to be completed. For future implementation of the use of *Cancer and You*, nurses suggested educating multiple patients as a group rather than as individual patients to increase efficiency.

## DISCUSSION

Results of the pretest and post-test analyses indicate that there was a clinically and statistically significant increase in patient knowledge about cancer, treatment, and treatment adverse effects using a standard patient education tool. Patient feedback also indicates that they were satisfied with the standardized *Cancer and You* booklet and wanted to take the booklet home to share with family and friends. The booklet and standard approach were well received such that all patients who were interviewed recommended that other patients who were starting chemotherapy receive education using the booklet.

The purpose of this project was to improve the quality of patient education at BCCOE; a few limitations exist. Whereas measures were taken to train nurses to provide standardized education, the delivery and time of patient education may have varied from nurse to nurse and whether this had any effect on results is unconcluded. Patients surveyed here are both a small sample size and a small representation of patients who receive treatment at BCCOE; therefore, results cannot be generalized to all types of cancers, patient populations (adult and pediatric), or settings. Although there was a significant increase in patient knowledge and satisfaction during this project, a causal relationship cannot be made between the *Cancer and You* booklet and patient knowledge or satisfaction, as many factors were not controlled for in the analysis of patient responses.

Moving forward, nurses involved in this project will serve as leaders to educate other oncology nurses at BCCOE on how to conduct standardized patient education on cancer, treatment, and treatment adverse effects. As patients were nearly split 50:50 in their recommendation for the best time to receive the *Cancer and You* booklet, it was decided by the nurses and nursing leadership to continue with standardized education at the time of cancer treatment initiation as conducted for this project and reinforce education with subsequent cycles.

Given the feedback from nurses on the time required for education, possible future directions may include group education sessions or having nurses facilitate in-person question-and-answer sessions after viewing a video-recorded standard patient education session. Future steps also include keeping a stock of booklets available for patients to take home and review for themselves or share with family members.

As outlined previously, patient experiences and outcomes have been shown to be favorable with improved education about their disease and treatment.^3,4^ With integration of the *Cancer and You* booklet, BCCOE hopes to lead the region in patient education experience and offer a model for other LMICs. Use of *Cancer and You* has resulted in a higher level of satisfaction not only for patients but also nurses. Inspired by the positive response of adult oncology patients, the chief nursing officer at BCCOE has worked to adapt the quality improvement project in the pediatric oncology ward to transform the patient education experience for all patients with cancer at BCCOE.

## Data Availability

The following represents disclosure information provided by authors of this manuscript. All relationships are considered compensated. Relationships are self-held unless noted. I = Immediate Family Member, Inst = My Institution. Relationships may not relate to the subject matter of this manuscript. For more information about ASCO's conflict of interest policy, please refer to www.asco.org/rwc or ascopubs.org/jgo/site/misc/authors.html. **Employment:** One Medical No other potential conflicts of interest were reported.
